# Prof. Winters on Constipation in Children

**Published:** 1889-06

**Authors:** 


### Prof. Winters on Constipation in Children.

Prof. J. E. Winters, in a recent clinic on constipation in infants, said that if the constipation is in a nursing infant, look first to the mother's bowels. If she is costive (has a daily but not a free and full movement), give her belladonna or ipecac, with small doses of strychnia, as the costiveness is probably due to diminished secretions in the alimentary canal. Follow these drugs with a cold, salt-water abdominal bath, kneading the abdomen thoroughly. If the mother is constipated (does not have daily movements), add some mild cathartic to the above treatment; cascara sagrada and the mineral waters are good. The diet should not be restricted but should consist of a plentiful supply of vegetables, fruits, and farinaceous food. Excessive tea drinking by the mother is sometimes the cause of colic and constipation in the infant. In such a case, discontinue the tea absolutely and use milk, chocolate, gruel, and coffee in moderation. If the cause should not lie in the mother, turn your attention to the child, and begin treatment with the juice of fruits; that of oranges is preferable, and a child two days old can take the juice of one-fourth of an orange, twice daily; and this amount should be increased daily. If this is not effectual try grape juice, or the meats of stewed prunes and cherries. The latter are especially good. The cold, salt-water abdominal bath should be used for the mother in connection with this treatment of the infant. If it does not act give an injection of glycerine (f℥ss-j) daily at a definite hour. It acts on the circulation and increases the secretion of the bowel. In addition to the injection, to every bottle of milk add a fluid drachm of glycerine, or a piece of fresh unsalted butter as large as a hazel nut. Never use castor oil or rhubarb. If drugs are necessary begin with a small dose of ipecac (gtt. ij-v), especially when the movements are hard and dry. Also use small doses of nux vomica and belladonna. If a cathartic becomes necessary, cascara sagrada is good. It does not lead to constipation and does not lose its effect by continual use. Begin with a small dose and increase it until there is a good movement daily, and then decrease it until it can be discontinued. Use fruit and glycerine injections with it. The use of opium by unscrupulous nurses and mothers is a frequent cause of constipation.

